# Detection of antibodies against H5 subtype highly pathogenic avian influenza viruses in multiple raccoons in Tokachi District, Hokkaido, Japan, from 2022 to 2023

**DOI:** 10.1016/j.virusres.2024.199515

**Published:** 2024-12-24

**Authors:** Minami Komami, James G. Komu, Yuki Ishiguro, Motoki Sasaki, Sachiko Matsuda, Dulamjav Jamsransuren, Vuong Nghia Bui, Yohei Watanabe, Kunitoshi Imai, Haruko Ogawa, Yohei Takeda

**Affiliations:** a Department of Veterinary Medicine, Obihiro University of Agriculture and Veterinary Medicine, 2-11 Inada, Obihiro, Hokkaido 080-8555, Japan; b Graduate School of Animal and Veterinary Sciences and Agriculture, Obihiro University of Agriculture and Veterinary Medicine, 2-11 Inada, Obihiro, Hokkaido 080-8555, Japan; c Department of Medical Laboratory Sciences, College of Health Sciences, Jomo Kenyatta University of Agriculture and Technology, Nairobi P.O. Box 62000-00200, Kenya; d Research Center for Global Agromedicine, Obihiro University of Agriculture and Veterinary Medicine, 2-11 Inada, Obihiro, Hokkaido 080-8555, Japan; e Virology Department, National Institute of Veterinary Research, Hanoi 100000, Vietnam; f Department of Infectious Diseases, Kyoto Prefectural University of Medicine, Kyoto 602-8566, Japan; g Department of Virology, The JIKEI University School of Medicine, Tokyo 105-8461, Japan

**Keywords:** H5 subtype highly pathogenic avian influenza virus, Clade 2.3.4.4b, Raccoon, Seroprevalence

## Abstract

In recent years, infection cases of H5 subtype highly pathogenic avian influenza viruses (HPAIVs) in wild mammals have increased globally. To obtain recent epidemiological information regarding influenza A virus (IAV) infection in raccoons (*Procyon lotor*), the prevalence of anti-IAV antibodies in sera was analyzed among raccoons captured in Tokachi District, Hokkaido, Japan, from 2019 to 2023. Screening of serum samples using enzyme-linked immunosorbent assay and agar gel precipitation test detected anti-IAV antibodies in 5 of 114 (4.4 %) raccoons. All positive sera were from raccoons captured from 2022 to 2023. The hemagglutination inhibition test revealed that all five serum samples contained anti-H5 subtype HPAIV antibodies, and one also contained anti-H1 subtype antibodies. The neuraminidase inhibition test revealed that all five sera contained anti-N1 subtype antibodies, and one also contained anti-N8 subtype antibodies. In the virus neutralization test, these five sera showed stronger neutralization activity against the H5 subtype clade 2.3.4.4b HPAIV strain recently circulating worldwide compared to the old H5 HPAIV strain isolated in Japan in 2007. These findings suggested that raccoons could be involved in the circulation of H5 HPAIVs in nature.

## Highlights

• Seroprevalence against IAV was evaluated in raccoons captured in Tokachi District.
• Five sera from raccoons captured from 2022 to 2023 were anti-IAV antibody-positive.
• All positive sera contained anti-H5 and -N1 antibodies.
• All positive sera showed strong reaction against H5 clade 2.3.4.4b HPAIV.
• One seropositive sample also contained anti-H1 and -N8 antibodies.

## 1 Introduction

Because the highly pathogenic avian influenza virus (HPAIV) causes fatal acute systemic diseases in gallinaceous poultry and extensive damage to the poultry industry worldwide (Nuñez and Ross, 2019), the spread of the virus is a critical global issue. In Japan, there have been repeated outbreaks of HPAI since 2004. In the past few years, the detection rate of H5 subtype HPAIVs in wild birds and the HPAI outbreaks in poultry farms have increased markedly. During the 2021–2022 season, 107 infection cases of H5 subtype HPAIVs, including H5N8 and H5N1 strains, were identified in wild birds, and ∼1.9 million poultry were culled; during the 2022–2023 season, 242 infection cases of H5 subtype HPAIVs, mainly H5N1 strains, were identified in wild birds, and >17.0 million poultry were culled (Ministry of Agriculture, Forestry and Fisheries, 2024). The A/goose/Guangdong/1/96 (H5N1)-lineage HPAIVs have evolved dramatically into 10 genetically different clades (clades 0–9) and their subclades (Gavin and Ruben, 2014). Since the first emergence of the H5 subtype clade 2.3.4.4b HPAIV in China in 2013 (Lee et al., 2017), viruses belonging to this subclade have spread globally, causing genetic reassortments with other strains, and have become dominant in various regions in Asia, Europe, Africa, and Americas (Charostad et al., 2023).

In AIV transmission to poultry, there are multiple possible routes, such as a fecal-oral route from wild birds to poultry (World Organization for Animal Health, 2016) and an indirect contact route via humans, insects, vehicles, and equipment (Globig et al., 2018; Napp et al., 2018; World Organization for Animal Health, 2016). In addition, possible virus transmission via dust, which includes feather epithelium shedding AIV, is nonnegligible (Gaide et al., 2023). Another likely route through which AIVs enter poultry farms might involve wild mammals. Although the clear evidence of AIV transmission from wild mammals to poultry is poor, cases of AIV-susceptible wild mammals invading farms have been reported and thus should be potential risk factors (Root and Shriner, 2020; Yamaguchi, 2016). Many HPAIV detection cases in mammals have been reported. Among these cases, H5N1 clade 2.3.4.4b HPAIVs have been detected in many mammal species, including badger, black bear, bobcat, coyote, dolphin, ferret, fisher cat, fox, lynx, mink, opossum, otter, pig, polecat, porpoise, raccoon, seal, and skunk (World Health Organization, 2022). Charostad et al. (2023) summarized many H5N1 HPAIV detection and outbreak cases in mammals from 2022 to 2023. For example, infectious H5N1 HPAIVs were isolated in a debilitated Japanese raccoon dog and a dead Ezo red fox in Hokkaido, Japan, in March 2022 (Hiono et al., 2023); outbreak and mass deaths by H5N1 HPAIV infection among seals were observed in the United States from June to July 2022 (Puryear et al., 2023); the outbreak by H5N1 HPAIVs occurred in a large-scale mink farm in Spain in October 2022 (Agüero et al., 2023); and the deaths of sea lions and a dolphin, which began in November 2022, were reported in Peru, and H5N1 HPAIVs were detected from these animals (Leguia et al., 2023). In all four reports, clade 2.3.4.4b viruses were detected. In the mink case in Spain, the T271A amino acid substitution in viral polymerase basic 2 protein (PB2), which was reported to increase influenza A virus (IAV) polymerase activity in mammalian cells (Bussey et al., 2010), was detected (Agüero et al., 2023). In sea lion cases in Peru, the detected IAVs had D701N amino acid substitution on PB2 (Leguia et al., 2023), one of the adaptation markers to mammals (Li et al., 2005). In another case, PB2 E627K substitution, which is a mammalian adaptation marker, was accelerated in a Tibetan black bear infected with H5N1 clade 2.3.4.4b HPAIV transmitted from a bird (Bessière et al., 2024). These reports indicated the possible risk of the emergence and spread of mammalian-adapted IAVs in wild mammals and highlighted the importance of further studies to clarify the role of these animals in HPAIV circulation in nature.

The raccoon (*Procyon lotor*) is an introduced animal species in Japan that has expanded its habitat area after escaping from a zoo and irresponsible abandonment or neglect by pet owners (Ikeda et al., 2004). It is designated as a specified invasive alien species in the country. Raccoons’ reproductive capacity is high, and their habitat area is large. Due to their omnivorous nature, they have direct impacts on the agricultural, forestry, and fisheries industries. Raccoons can carry pathogens that cause zoonotic diseases, such as *Baylisascaris procyonis* and rabies virus (Wandeler and Salsberg, 1999; Yeitz et al., 2009). The Japanese law on the Prevention of Adverse Ecological Impacts Caused by Designated Invasive Alien Species allows the capture and eradication of raccoons. Previous serological studies suggested the establishment of infections of IAVs, including H5 HPAIVs and other subtype IAVs in raccoons (Hall et al., 2008; Horimoto et al., 2011). In our previous serosurveillance study on raccoons captured from 2009 to 2012 in eastern Japan, anti-IAV antibodies were detected in 12 of 634 serum samples (Yamaguchi et al., 2014). These findings suggested that raccoons are likely involved in IAV circulation in nature. However, very little is known about the actual infection status of raccoons during recent years when HPAI outbreaks have repeatedly occurred in poultry and many HPAIVs have been detected in wild birds. This study aimed to obtain recent epidemiological information on IAV infection in raccoons captured in Tokachi District from 2019 to 2023 by collecting and analyzing serum samples for anti-AIV antibodies using serological methods.

## 2 Materials and methods

### 2.1 Raccoon samples

Blood samples (*n* = 114; 91 males, 22 females, 1 unknown) were collected from raccoons captured under the official eradication program in Tokachi District from 2019 to 2023. The ages of the raccoons were estimated based on whether the root apical foramen of the canine teeth was open or closed (Grau et al., 1970) and degree of cranial suture closure (Junge and Hoffmeister, 1980). Considerably, the estimated age was divided into three stages: Raccoons were considered (1) aged <12 months old and “juvenile” based on cranial suture assessment and open apical foramina; (2) approximately 12 months old as indicated by cranial suture assessment, regardless of the apical foramen status; and >12 months old based on cranial suture assessment and closed apical foramina.

Sera separated from clotted whole blood were stored at −30 °C until analyses. Before the analyses, test sera were treated with a receptor-destroying enzyme (RDE; Denka Seiken, Tokyo, Japan) to remove nonspecific inhibitors for serological tests.

Nasal swabs (*n* = 236) were also collected from captured raccoons from 2017 to 2023 to attempt to isolate infectious IAVs. These nasal swabs were soaked in virus transfer medium comprising Dulbecco's modified Eagle's medium (DMEM; Nissui Pharmaceutical, Tokyo, Japan) with 0.5 % bovine serum albumin (BSA; FUJIFILM Wako Pure Chemical, Osaka, Japan), 1 mg/mL kanamycin (Meiji Seika Pharma, Tokyo, Japan), 100 μg/mL of gentamycin (MSD Japan, Tokyo, Japan), and 5 μg/mL amphotericin B (Bristol–Myers Squibb, New York, NY, USA). The swabs were reacted with antibacterial and antifungal agents present in the virus transfer medium for 2 h at room temperature (RT). Subsequently, the mixtures were centrifuged for 10 min, and the supernatants were collected and stored at −80 °C until used for analysis.

### 2.2 Enzyme-linked immunosorbent assay (ELISA)

ELISA was performed to confirm the presence of anti-IAV nucleoprotein (NP) antibodies in serum samples. The solutions of recombinant NP, which was produced by introducing the NP gene of A/chicken/Yokohama/aq55/01 (H9N2) into *Escherichia coli*, as described previously (Horie et al., 2009), were prepared by diluting with carbonate–bicarbonate buffer (pH 9.6). The recombinant NP solution (5 or 10 μg/mL) was added in Clear Flat-Bottom Immuno Nonsterile 96-Well Plates (Thermo Fisher Scientific, Waltham, MA, USA) at 50 μL/well for antigen coating. After blocking with phosphate-buffered saline (PBS; pH 7.4) containing 1 % BSA (BSA/PBS), RDE-treated test sera serially diluted to twofold starting at 1:100 with BSA/PBS were added at 50 μL/well. The plates were incubated at RT for 1 h. As the secondary antibody, 0.1 μg/mL of Purified Recomb® Protein A/G, Peroxidase Conjugated (Pierce Chemical, Rockford, IL, USA) diluted with BSA/PBS was added at 50 μL/well. The plates were incubated at RT for 1 h. The TMB Substrate Reagent Set (BD Biosciences, San Diego, CA, USA) was added at 100 μL/well. The plates were incubated at RT for 15 min. Finally, 50 μL/well of 2 N H_2_SO_4_ was added to stop the reaction. Absorbance (optical density) at 450 nm (OD_450__nm_) was measured using GENios Pro (Tecan Australia, Melbourne, Australia).

### 2.3 Agar gel precipitation (AGP) test

Serum samples with relatively higher absorbance at OD_450__nm_ than other samples in ELISA (in principle, >0.6 value for the 1:400 diluted sera) were further analyzed to evaluate the presence of anti-IAV antibodies using the AGP test, which is less sensitive but more specific to antibody detection than NP-based indirect ELISA (Comin et al., 2013). AGP tests were performed as described previously (Beard, 1970; Yamaguchi et al., 2014). Briefly, the antigen for AGP was prepared by collecting chorioallantoic membranes from 10-day-old grown chicken eggs infected with H3N8 subtype strain A/budgerigar/Aichi/97, which was kindly provided by the National Institute of Animal Health (NIAH; Ibaraki, Japan). The membranes collected were cut into small pieces and homogenized in PBS. The homogenates were treated with 0.1 % formalin to inactivate the virus. The 1 % agar gel with 0.1 % NaN_3_ was poured onto the plate to solidify it. Wells were created using a gel puncher. RDE-treated test sera (1:4 dilution) and the antigen solution were poured into the prepared wells, respectively. A raccoon serum sample confirmed seropositive in our previous study (Yamaguchi et al., 2014) was used as the positive control serum. After 2 days of incubation at RT, the presence or absence of a sedimentation line was observed between the well containing each test sera or positive control serum and the well containing the antigen solution.

### 2.4 Hemagglutination inhibition (HI) test

HI tests were performed according to the World Health Organization (WHO) manual on animal influenza diagnosis and surveillance (World Health Organization, 2002) using 0.5 % chicken red blood cell suspension. RDE-treated test sera were twofold serially diluted in PBS starting from 1:8 or 1:10. The 25 μL of these diluted sera were mixed with four hemagglutination units/25 μL of the viruses and incubated at RT for 30 min. The 50 μL chicken red blood cell suspension was added to the mixture and incubated for 25 min. The HI titer was determined to have the highest dilution fold of the serum, showing a complete inhibition of hemagglutination.

The following reference strains of viruses provided by the OIE Reference Laboratory for AIV at Hokkaido University (Sapporo, Japan) were used for the HI test: A/swine/Hokkaido/1/81 (H1N1), A/duck/Hong Kong/278/78 (H2N9), A/duck/Hokkaido/5/77 (H3N2), A/chicken/Czechoslovakia/56 (H4N6), A/duck/Hong Kong/820/80 (H5N3), A/shearwater/South Australia/1/72 (H6N5), A/duck/Hong Kong/301/78 (H7N2), A/turkey/Ontario/6118/67 (H8N4), A/duck/Hong Kong/448/78 (H9N2), A/chicken/Germany/N/49 (H10N7), A/duck/England/1/56 (H11N6), A/duck/Alberta/60/76 (H12N5), A/gull/Maryland/704/77 (H13N6), A/mallard/Astrakhan/263/82 (H14N5), and A/duck/Australia/341/83 (H15N8).

Other H5 subtype HPAIVs isolated in Japan over the past two decades were also used to examine the HI activity of test sera. The viruses used for the HI test were as follows: A/chicken/Miyazaki/K11/07 (H5N1, clade 2.2) and A/chicken/Yamaguchi/7/04 (H5N1, clade 2.5) provided by the NIAH and A/white-tailed eagle/Japan/OU-1/2022 (H5N1, clade 2.3.4.4b) isolated in our laboratory (Komu et al., 2023).

### 2.5 Neuraminidase (NA) inhibition (NAI) test

NA and NAI tests were performed according to the WHO manual (World Health Organization, 2002). The NA activity of the target viruses was measured to determine the optimal virus dilution, giving an absorbance of 0.5 at OD_549__nm_ (1 unit) in NAI tests. Then, 1 unit/10 μL of the virus solution was mixed with an equal volume of serum samples, which were twofold serially diluted starting from 1:20. Instead of serum samples, PBS was used as the negative control. As positive controls, sera against A/swine/Hokkaido/1/81 strain, which is the H1N1 subtype reference strain, and A/white-tailed eagle/Hokkaido/22-RU-WTE-2/2022 strain (H5N1, clade 2.3.4.4b), which was recently isolated (Isoda et al., 2022) and kindly provided by the OIE Reference Laboratory for AIV at Hokkaido University, were used.

After these mixtures were incubated at RT for 30–60 min, 20 μL fetuin solution (Sigma, St. Louis, MO, USA) was added. Tubes containing the mixtures were incubated at 37 °C for 18–20 h and cooled to RT. The periodate reagent was added to each tube, shaken, and incubated at RT for 20 min. The reaction was stopped by the addition of an arsenite reagent. The thiobarbituric acid reagent was added to each tube and incubated at 100 °C for 15 min. After cooling the tubes on ice, 1-butanol was added to each tube. The tubes were centrifuged for 10 min. The amount of liberated sialic acid was chemically determined by measuring the color developed in a spectrophotometer at OD_549__nm_. NAI titer was determined to have the highest dilution fold of test serum, showing 50 % inhibition of NA activity.

The reference strains of viruses used for the NAI test included A/swine/Hokkaido/1/81 (H1N1), A/duck/Hong Kong/301/78 (H7N2), A/duck/Hong Kong/820/80 (H5N3), A/turkey/Ontario/6118/67 (H8N4), A/shearwater/South Australia/1/72 (H6N5), A/chicken/Czechoslovakia/56 (H4N6), A/chicken/Germany/N/49 (H10N7), A/duck/Australia/341/83 (H15N8), and A/duck/Hong Kong/278/78 (H2N9). A/chicken/Kumamoto/1-7/2014 (H5N8, clade 2.3.4.4c) provided by the NIAH and the isolate A/white-tailed eagle/Japan/OU-1/2022 (H5N1, clade 2.3.4.4b) were used.

### 2.6 Virus neutralization (VN) test

VN tests were conducted according to the WHO manual (World Health Organization, 2002). Madin–Darby canine kidney (MDCK) cells were cultivated in DMEM supplemented with 10 % fetal bovine serum and 2 mM l-glutamine (FUJIFILM Wako Pure Chemical) in a humidified incubator with 5 % CO_2_ at 37 °C. The stock solutions of viruses were inoculated to MDCK cells. The 50 % tissue culture infectious dose (TCID_50_) of each virus was determined using the Behrens–Kärber method (Kärber, 1931). Then, 200 TCID_50_/100 μL of each virus solution was prepared by diluting the stock solution with virus growth medium composed of DMEM supplemented with 0.2 % BSA, 0.01 % glucose (FUJIFILM Wako Pure Chemical), 0.15 % NaHCO_3_ (FUJIFILM Wako Pure Chemical), and 2.5 mM HEPES (FUJIFILM Wako Pure Chemical). Trypsin was added in the virus growth medium with a final concentration of 0.0063 % when low pathogenic AIV (LPAIV) was inoculated into cells, whereas it was not added for HPAIV. RDE-treated test sera were twofold serially diluted from 40- to 1280-fold. These diluted sera were mixed with an equal amount of virus solutions (200 TCID_50_/100 μL) and incubated at 37 °C for 2 h. The mixture was added to MDCK cells in a 96-well microplate and incubated for 2 h. After washing twice, the virus growth medium was added to cells and incubated for 4 days. The cytopathic effect was observed. The VN titer was defined as the highest dilution of the test serum that completely suppressed the cytopathic effect. Viruses used for the VN tests were as follows: A/duck/Hong Kong/820/80 (H5N3), which is LPAIV, and A/chicken/Miyazaki/K11/07 (H5N1, clade 2.2) and A/white-tailed eagle/Japan/OU-1/2022 (H5N1, clade 2.3.4.4b), which are HPAIVs. The antiserum against A/duck/Hong Kong/820/80 (H5N3) was provided by the OIE Reference Laboratory for AIV at Hokkaido University and used as a control serum against the H5-subtype reference strain.

### 2.7 Attempt of IAV isolation

For virus isolation, 0.1 mL of nasal swab samples (individual or pooled samples) were inoculated via the allantoic route into two 10-day-old embryonated chicken eggs, incubated at 37 °C for 72 h. The allantoic fluids (AFs) were collected from the eggs, and their hemagglutination activities were evaluated using the hemagglutination test (World Health Organization, 2002). Hemagglutination-negative AFs were further passaged once in eggs. When AFs showed hemagglutination activity, real-time RT-PCR targeting IAV gene was performed to evaluate whether IAV was isolated or not. Briefly, RNAs were extracted from AFs using a QIAamp Viral RNA Kit (QIAGEN, Venlo, The Netherlands) per the manufacturer's instructions. The cDNAs were synthesized from the extracted RNAs using FastGene Scriptase II (Nippon Genetics, Tokyo, Japan) under the following conditions: 25 °C for 10 min, 42 °C for 60 min, and 85 °C for 5 min. Real-time PCR targeting the IAV matrix gene was performed, as described previously (Runstadler et al., 2007).

## 3 Results

### 3.1 Detection of anti-IAV antibody-positive raccoon sera

**Fig. 1:**
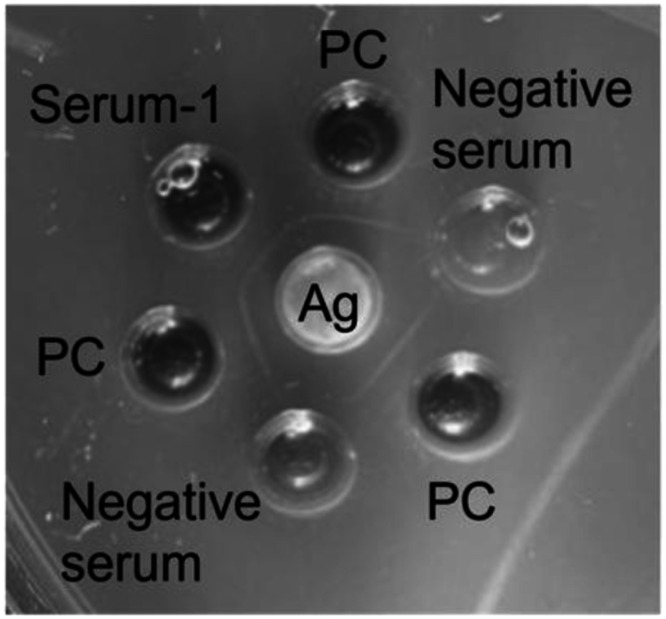
**Representative photo of the AGP test.** Results of Serum-1 presented as representative findings of the AGP test; a sedimentation line was observed between the wells containing antigen and Serum-1. PC: positive control serum; Ag: antigen.

To detect anti-IAV antibodies in sera, ELISA detecting anti-NP antibodies and AGP tests detecting the antibodies against conserved proteins of IAVs such as NP and matrix protein were performed. The two assays targeted antibodies against IAV common antigens essentially present in all IAVs irrespective of hemagglutinin (HA) and NA subtypes. In ELISA and AGP tests, sera from five raccoons captured in 2022 or 2023 were seropositive (Table 1, Fig. 1). All raccoons were male. The locations of these captured seropositive raccoons were away from poultry farms that experienced HPAI outbreaks. No information has been obtained on any wild bird habitats near these locations.

**Table 1:** Anti-IAV antibody-positive raccoon sera in ELISA and AGP tests.

Sample ID	Captured yearand month	Estimated age	OD_450__nm_ of the 1:400 diluted serum analyzed by ELISA^a^
Serum-1	2022 Apr	>12 months old	1.62 ± 0.22
Serum-2	2022 Sep	>12 months old	0.89 ± 0.33
Serum-3	2023 May	Unknown	1.18 ± 0.15
Serum-4	2023 Jun	>12 months old	0.90 ± 0.15
Serum-5	2023 Jun	Approximately 12 months old	1.30 ± 0.26

a Each ELISA was repeated twice for all samples. The mean OD value ± standard deviation of the two replicates is shown. The OD values for all samples in both replicates were >0.6.

### 3.2 Determination of the HA subtype of IAVs recognized by anti-IAV antibody-positive raccoon sera

The five raccoon sera positive for anti-IAV antibodies in ELISA and AGP were forwarded to the HI test for HA subtyping using H1–15 subtype reference strains. In the HI test using the H1 subtype reference strain, A/swine/Hokkaido/1/81, one (Serum-1) of the five sera showed HI activity against the H1 subtype virus with the HI titer 64. In the HI test using the H5 subtype reference strain, A/duck/Hong Kong/820/80 (LPAIV) strain, two (Serum-3 and -5) of the five sera showed HI activity against the H5 subtype virus with HI titers 10–20. All five sera were further analyzed by the HI test using three different H5N1 subtype HPAIV strains, A/chicken/Miyazaki/K11/07 (clade 2.2), A/chicken/Yamaguchi/7/04 (clade 2.5), and A/white-tailed eagle/Japan/OU-1/2022 (clade 2.3.4.4b). HI test results using three HPAIV strains indicated that all five raccoon sera showed HI activity against the H5 subtype HPAIV A/white-tailed eagle/Japan/OU-1/2022 (clade 2.3.4.4b), which gave HI titers 10 to 80. Strikingly, the other two strains of H5 subtype HPAIVs, A/chicken/Miyazaki/K11/07 (clade 2.2) and A/chicken/Yamaguchi/7/04 (clade 2.5), reacted only with the two serum samples (Serum-3 and -5), similar to results with the H5 subtype reference strain, with similar HI titers 10 to 20 (Table 2). In summary, all five serum samples were confirmed to contain anti-H5 antibodies. The seroprevalence against H5 subtype IAV was 4.4 % (5 positive in 114 samples). The sample size adequacy for estimating the seroprevalence was evaluated using the web tool EPITOOLS (https://epitools.ausvet.com.au/oneproportion). The inputs were as follows: estimated true proportion: 0.04; desired precision (+/−): 0.05; confidence level: 0.95; and population size (for finite populations): 114. The calculated required sample size for specified inputs was 40; thus, herein, the sample size was sufficient. Among Serum-1–5, one sample (Serum-1) also contained an antibody against H1 subtype IAV. None of the tested raccoon sera exhibited HI activity against H2–4 and H6–15 subtype reference strains.

**Table 2:** HI titers of test sera against the H1 subtype IAV strain and multiple H5 subtype strains.

Sample ID	HI titers against H1 and H5 subtype IAV strains				
	Target virus: H1 subtype strain	Target virus: H5 subtype strain			
	A/swine/Hokkaido/1/81 H1N1^a^^,^*	A/duck/Hong Kong/820/80 H5N3^b^^,^^c^^,^*	A/chicken/Miyazaki/K11/07 H5N1^d^ (clade 2.2)	A/chicken/Yamaguchi/7/04 H5N1^d^ (clade 2.5)	A/white-tailed eagle/Japan/OU-1/2022 H5N1^d^^,^* (clade 2.3.4.4b)
Serum-1	64	<10	<10	<10	20
Serum-2	<8	<10	<10	<10	10
Serum-3	<8	10	20	20	80
Serum-4	<8	<10	<10	<10	20
Serum-5	<8	20	20	20	80

a H1 subtype reference strain.

b H5 subtype reference strain.

c LPAIV strain.

d HPAIV strain.

⁎ The test was repeated twice, and the results of the first experiment are presented. The replicate test results are shown in Supplementary Table 1.

### 3.3 Determination of the NA subtype of IAVs recognized by anti-IAV antibody-positive raccoon sera

The NAI test was performed using A/swine/Hokkaido/1/81 (H1N1) and A/white-tailed eagle/Japan/OU-1/2022 (H5N1) strains.

In the NAI test using A/swine/Hokkaido/1/81 (H1N1) strain, the N1 subtype strain isolated in 1981, the NAI titer was below the detection limit (<20) in one serum sample (Serum-2), whereas those of other four serum samples (Serum-1, -3, -4, and -5) ranged from 20 to 160. The NAI test using antisera against A/swine/Hokkaido/1/81 (H1N1) and A/white-tailed eagle/Hokkaido/22-RU-WTE-2/2022 (H5N1), which were used as positive control sera, showed their NAI titers against A/swine/Hokkaido/1/81 (H1N1) with 160 to 320 and 20 to 40, respectively.

In the NAI test using A/white-tailed eagle/Japan/OU-1/2022 (H5N1), a recent N1 subtype HPAIV strain, NAI antibodies were detected in all five serum samples. The five serum samples showed higher NAI titers, ranging from 640 to >2560, compared to those against A/swine/Hokkaido/1/81 (H1N1), ranging from <20 to 160. Antisera against A/swine/Hokkaido/1/81 (H1N1) and A/white-tailed eagle/Hokkaido/22-RU-WTE-2/2022 (H5N1) showed their NAI titers against A/white-tailed eagle/Japan/OU-1/2022 (H5N1) with 320 to 640 and >2560, respectively (Table 3).

**Table 3:** NAI titers of test sera against N1 and N8 subtype AIVs.

Sample ID	NAI titers against two strains of N1 subtype IAVs		NAI titers against two strains of N8 subtype IAVs	
	Target virus: A/swine/Hokkaido/1/81 (H1N1)^a^	Target virus: A/white-tailed eagle/Japan/OU-1/2022 (H5N1)^b^	Target virus: A/duck/Australia/341/83 (H15N8)^c^	Target virus: A/chicken/Kumamoto/1-7/2014 (H5N8)^b^
Serum-1	80–160^f^	1280–2560	20–40	20–40
Serum-2	<20	>2560	n.t.^g^	n.t.
Serum-3	40–80	640–1280	n.t.	n.t.
Serum-4	20–40	640–1280	n.t.	n.t.
Serum-5	20–40	>2560	n.t.	n.t.
Anti-H1N1 serum^d^	160–320	320–640	n.t.	n.t.
Anti-H5N1 serum^e^	20–40	>2560	n.t.	n.t.

a N1 subtype reference strain.

b HPAIV strain.

c N8 subtype reference strain.

d Positive control: antiserum against A/swine/Hokkaido/1/81 (H1N1), N1 subtype reference strain.

e Positive control: antiserum against A/white-tailed eagle/Hokkaido/22-RU-WTE-2/2022 (H5N1).

f It means that NAI titer was higher than 80 and lower than 160.

g Not tested.

Because Serum-1 showed HI activity against two HA subtype IAVs, suggesting that this raccoon had been infected with multiple IAV strains, this sample was subjected to additional NAI testing using N2 to N9 subtype reference strains. The NAI antibody was detected against A/duck/Australia/341/83 (H15N8) with titers 20 to 40. Furthermore, this sample was forwarded to the NAI test using A/chicken/Kumamoto/1-7/2014 (H5N8). The NAI titer against this strain was also 20 to 40 (Table 3). Therefore, all five serum samples contained the anti-N1 antibody, and Serum-1 also contained the anti-N8 antibody (Table 4).

**Table 4:** HA and NA subtypes of IAVs infected to the raccoons tested in this study based on HI and NAI tests.

Sample ID	HA subtype of the infected virus	NA subtype of the infected virus
Serum-1	H1, H5	N1, N8
Serum-2	H5	N1
Serum-3	H5	N1
Serum-4	H5	N1
Serum-5	H5	N1

### 3.4 Evaluation of the VN activity of anti-IAV antibody-positive sera against H5 subtype AIVs

To analyze the neutralizing activity of test sera against multiple H5 subtype AIVs, the VN test was performed. All five sera did not show any neutralizing activities to A/duck/Hong Kong/820/80 (H5N3, LPAIV), the H5 subtype reference strain, and A/chicken/Miyazaki/K11/07 (H5N1, HPAIV, clade 2.2). In contrast, all five sera showed high VN titers to A/white-tailed eagle/Japan/OU-1/2022 (H5N1, HPAIV, clade 2.3.4.4b); the VN titers were 320 to 640. Comparing these five sera, the VN titers of the antiserum against A/duck/Hong Kong/820/80 (H5N3) to the three H5 subtype strains were also analyzed. Contrary to the results for the five raccoon sera, the VN titer of this antiserum was >1280, 80, and <40 to A/duck/Hong Kong/820/80 (homologous strain), A/chicken/Miyazaki/K11/07, and A/white-tailed eagle/Japan/OU-1/2022, respectively (Table 5).

**Table 5:** VN titers of test sera against H5 subtype AIVs.

Sample ID	VN titers against H5 subtype AIVs		
	Target virus: A/duck/Hong Kong/820/80 H5N3^a^^,^^b^	Target virus: A/chicken/Miyazaki/K11/07 H5N1^c^ (clade 2.2)	Target virus: A/white-tailed eagle/Japan/OU-1/2022 H5N1^c^ (clade 2.3.4.4b)
Sample-1	<40	<40	640
Sample-2	<40	<40	320
Sample-3	<40	<40	640
Sample-4	<40	<40	320
Sample-5	<40	<40	320
Anti-duck/Hong Kong serum^d^	>1280	80	<40

a H5 subtype reference strain.

b LPAIV strain.

c HPAIV strain.

d Antiserum against A/duck/Hong Kong/820/80 (H5N3).

### 3.5 No isolation of IAV from nasal swab samples

The isolation of IAV from nasal swabs (*n* = 236) from raccoons captured between 2017 and 2023 were attempted. Five raccoons were seropositive against AIVs (Serum-1–5). Although several AFs harvested from eggs inoculated with the nasal swab samples showed weak hemagglutination activity, the IAV M genes were not detected in these AFs.

## 4 Discussion

Anti-IAV antibodies were detected in five raccoon serum samples among 114 samples collected from 2019 to 2023. All these sera showed HI activity against the H5 subtype HPAIV (Table 1, Table 2). This result suggested that multiple infection cases of H5 HPAIVs occurred in raccoons in the Tokachi District during and/or before this period. All raccoons were found alive in captured traps in which foods were set. During that time, these animals no longer carried infectious viruses. These findings implied that these raccoons likely developed the acquired immunity without getting fatal outcomes after HPAIV infection and could move to search for foods after recovery. In a previous study, raccoons confirmed to be experimentally infected with H4N6 subtype AIV and H3N2 subtype human IAV did not show clinical symptoms after infection with either virus (Hall et al., 2008). Regarding H5 subtype HPAIV infection cases, H5N1 clade 2.3.4.4b viruses were detected in four raccoons in the United States in 2022. Three raccoons were juveniles, and one was adult. Among the three juveniles, two were found dead, and the other was alive but showed neurological signs of diseases and ataxia. The one adult raccoon was also alive but showed neurological disorders (Elsmo et al., 2023). In another report, sera collected from healthy raccoons captured from 2005 to 2009 in Japan showed neutralizing activity against H5 subtype HPAIVs (Horimoto et al., 2011). These studies and our findings suggested that H5 subtype HPAIV infection in raccoons may cause fatal diseases, whereas in other cases, infections might be mild, and raccoons may recover and survive. Those surviving raccoons possibly spread the virus through their movements.

The HI and VN test results for raccoon sera showed higher antibody titers against the H5 clade 2.3.4.4b HPAIV strain isolated in 2022 (A/white-tailed eagle/Japan/OU-1/2022) than those against other H5 subtype HPAIVs and a LPAIV isolated in previous years (Table 2, Table 5). In Japan, clade 2.3.4.4b has been the dominant clade of H5 subtype HPAIVs, commonly detected from poultry and wild birds in recent years (Ministry of Agriculture, Forestry and Fisheries, 2024). The antigenicity of H5 viruses of clade 2.3.4.4 was confirmed to differ from those of H5 viruses from other clades (Ohkawara et al., 2017). Therefore, it is speculated that the five raccoons from which anti-H5 antibodies were detected in this study had been infected with HPAIV of clade 2.3.4.4, possibly clade 2.3.4.4b.

In the NAI test, the NA activity of A/white-tailed eagle/Japan/OU-1/2022 (H5N1) was more strongly inhibited by five raccoon sera compared to the antiserum against the N1 subtype reference strain, A/swine/Hokkaido/1/81 (H1N1; Table 3). Although the nucleotide substitution speed in the NA gene is slower than that of the HA gene, the NA gene also showed genetic evolution over the past few decades (Westgeest et al., 2012). Consistent with the previous finding, the NAI test results in this study suggested that the antigenicity of NA proteins of N1 subtype IAVs that have infected raccoons in recent years was different from that of the previous A/swine/Hokkaido/1/81 (H1N1) strain.

In our previous study, the seroprevalence of anti-IAV antibodies among raccoons captured in eastern Japan from 2009 to 2012 was 1.9 % (12 positive in 634 samples) (Yamaguchi et al., 2014). Among these IAV-positive samples, two sera containing anti-H5 subtype IAV antibodies were detected at a prevalence of 0.3 % (2 positive in 634 samples). In another study, the seroprevalence of anti-H5 subtype IAV antibodies was 0.9 % (10 positive in 1088 samples) among raccoons captured from 2005 to 2009 in eastern and western Japan (Horimoto et al., 2011). These percentages (0.3 % and 0.9 %) were much lower than that (4.4 %) of sera carrying anti-H5 HPAIV antibodies in raccoons captured in Tokachi District from 2019 to 2023. In the reports by Yamaguchi et al. (2014) and Horimoto et al. (2011), information on the specific region where raccoons were captured was not mentioned. Therefore, the possibility that the differences in geographic location and scale of the screening contributed to the difference in seroprevalence cannot be excluded from the two previous reports and this study. Nevertheless, because the number of H5Nx subtype HPAIV detection cases has increased in wild birds in Japan in the last 10 years (Ministry of the Environment, 2024), such an increase may critically correlate with the increase of seropositive cases in raccoons. In 2022, an H5N1 clade 2.3.4.4b HPAIV, A/white-tailed eagle/Japan/OU-1/2022, was isolated from a dead white-tailed eagle. The genome sequence of this strain showed high similarity with those of other H5 HPAIV strains circulating in Japan since 2021. The HI test revealed that the antigenicity of this strain's HA was quite different from H5 HPAIV strains isolated before 2007 (Komu et al., 2023). Such an antigenic change of the HA protein was similarly observed in HI and VN tests for anti-H5 antibody-positive raccoon sera tested in this study (Table 2, Table 5). Given that raccoons are omnivorous, they likely fed on dead wild birds infected with recently circulating H5 clade 2.3.4.4b HPAIVs.

Among the five seropositive samples, Serum-1 showed HI activity against H1 and H5 subtype IAVs (Table 2). This serum reacted with N1 and N8 subtype viruses in the NAI test (Table 3). These results suggested that this raccoon was previously infected with two or more IAV strains with different HA and NA subtypes. In Japan, the H5N8 subtype was dominantly circulating during the 2020–2021 season. The H5N1 subtype was dominant during the 2021–2022 and 2022–2023 seasons (Ministry of Agriculture, Forestry and Fisheries, 2024). Therefore, raccoons might have been infected with H5N1 and/or H5N8 subtype HPAIVs. In contrast, in our previous study, antibodies against the H1N1 virus were detected in four raccoons. The antibodies against the H1N8 virus were detected in one raccoon captured from 2009 to 2012 (Yamaguchi et al., 2014). Hence, the one raccoon (Serum-1) in this study might have been infected with H1N1 and/or H1N8 subtype IAVs. Although whether this H1 subtype virus was AIV or other animal species (including human)-derived IAV could not be clarified, Hall et al. (2008) reported that respiratory tissues of raccoons expressed avian- and human-type IAV receptors. Experimental infection indeed proved AIV and human-type IAV infections. This means that the potential virus transmission risk between a raccoon and a human in nature cannot be completely denied. In other important findings, multiple H5 HPAIV infection cases in domestic animals, including dairy cattle and a goat were reported in the United States in 2024. One H5 HPAIV infection case was confirmed in a human who had direct contact with a sick cow (Ly, 2024). Because raccoon intrusions into livestock farms have been confirmed and the animals eat livestock feed and posture grasses (Ikeda et al., 2004), it is possible that they come into contact with livestock. Although most mammalian hosts are considered dead-end hosts and may not be significantly involved in AIV spread, the potential risk of interspecies transmission of H5 HPAIV via raccoons should be considered.

## 5 Conclusion

Five raccoons in Tokachi District, Hokkaido, Japan, were infected with the H5 subtype HPAIVs during or before 2022 to 2023. According to serological assays, these viruses were presumed to be H5 subtype clade 2.3.4.4b HPAIVs circulating in wild birds and poultry in recent years. One raccoon was infected with multiple subtype IAVs, including H5 and H1 subtype viruses. These findings indicated the possibility that raccoons are involved in the spread and circulation of IAVs, including HPAIVs, in nature. Nevertheless, whether raccoons contribute to AIV transmission from wild birds to poultry and other mammals is still unknown. HPAIV outbreaks continue in many parts of the world and are causing serious damage globally. Continued epidemiological studies are required to clarify in more detail the role of raccoons in HPAIV circulation.

## Funding sources

This work was supported by JSPS KAKENHI [grant numbers 20KK0224].

## Data availability

Data will be made available on request.

## CRediT authorship contribution statement

**Minami Komami:** Writing – original draft, Visualization, Validation, Investigation. **James G. Komu:** Writing – review & editing, Visualization, Validation, Investigation. **Yuki Ishiguro:** Writing – review & editing, Validation, Investigation, Resources. **Motoki Sasaki:** Resources. **Sachiko Matsuda:** Validation, Investigation. **Dulamjav Jamsransuren:** Validation, Investigation. **Vuong Nghia Bui:** Writing – review & editing. **Yohei Watanabe:** Writing – review & editing, Project administration, Funding acquisition. **Kunitoshi Imai:** Writing – review & editing, Methodology. **Haruko Ogawa:** Writing – review & editing, Writing – original draft, Validation, Supervision, Resources, Project administration, Methodology, Funding acquisition, Conceptualization. **Yohei Takeda:** Writing – review & editing, Writing – original draft, Visualization, Validation, Supervision, Resources, Project administration, Methodology, Funding acquisition.

## Declaration of competing interest

The authors declare that they have no known competing financial interests or personal relationships that could have appeared to influence the work reported in this paper.

## Data Availability

No data was used for the research described in the article.

## Appendix Supplementary materials

Image, application 1
