# Supplementary material for: Detection of antibodies against H5 subtype highly pathogenic avian influenza viruses in multiple raccoons in Tokachi District, Hokkaido, Japan, from 2022 to 2023
Source: Virus Res. 2024 Dec 24;351:199515. doi: 10.1016/j.virusres.2024.199515 (PMC11732224; doi:10.1016/j.virusres.2024.199515)
Supplement: Supplementary file 1 [file mmc1.docx]

Supplementary materials for **Detection of antibodies against H5 subtype highly pathogenic avian influenza viruses in multiple raccoons in Tokachi District, Hokkaido, Japan, from 2022 to 2023**

Minami Komami^a^, James G. Komu^b,c^, Yuki Ishiguro^a^, Motoki Sasaki^a^, Sachiko Matsuda^d^, Dulamjav Jamsransuren^d^, Vuong Nghia Bui^e^, Yohei Watanabe^f,g^, Kunitoshi Imai^a^, Haruko Ogawa^a^, Yohei Takeda^a,d,^*

^a^ Department of Veterinary Medicine, Obihiro University of Agriculture and Veterinary Medicine, 2-11 Inada, Obihiro Hokkaido, 080-8555, Japan

^b^ Graduate School of Animal and Veterinary Sciences and Agriculture, Obihiro University of Agriculture and Veterinary Medicine, 2-11 Inada, Obihiro, Hokkaido 080-8555, Japan

^c^ Department of Medical Laboratory Sciences, College of Health Sciences, Jomo Kenyatta University of Agriculture and Technology, Nairobi P.O. Box 62000-00200, Kenya

^d^ Research Center for Global Agromedicine, Obihiro University of Agriculture and Veterinary Medicine, 2-11 Inada, Obihiro, Hokkaido 080-8555, Japan

^e^ Virology Department, National Institute of Veterinary Research, Hanoi 100000, Vietnam

^f^ Department of Infectious Diseases, Kyoto Prefectural University of Medicine, Kyoto 602-8566, Japan

^g^ Department of Virology, The JIKEI University School of Medicine, Tokyo 105-8461, Japan

* Corresponding author: Research Center for Global Agromedicine, Obihiro University of Agriculture and Veterinary Medicine, 2-11 Inada, Obihiro, Hokkaido 080-8555, Japan

E-mail address: ytakeda@obihiro.ac.jp (Y. Takeda).

**Supplementary Table 1. HI titers of test sera against the H1**

**and H5 subtype IAVs in the replicate experiment**

| Sample ID | HI titers against H1 and H5 subtype IAV strains | | |
| --- | --- | --- | --- |
|  | A/swine/  Hokkaido/1/81  H1N1^a^ | A/duck/Hong Kong/820/80  H5N3^b,c^ | A/white-tailed eagle/Japan/  OU-1/2022  H5N1^d^  (clade 2.3.4.4b) |
| Serum-1 | 80 | 10 | 40 |
| Serum-2 | <10 | 10 | 20 |
| Serum-3 | <10 | 10 | 80 |
| Serum-4 | <10 | 10 | 40 |
| Serum-5 | <10 | 10 | 80 |

^a^ H1 subtype reference strain

^b^ H5 subtype reference strain

^c^ LPAIV strain

^d^ HPAIV strain
